# Ancient DNA analysis of Indigenous rockfish use on the Pacific Coast: Implications for marine conservation areas and fisheries management

**DOI:** 10.1371/journal.pone.0192716

**Published:** 2018-02-13

**Authors:** Antonia T. Rodrigues, Iain McKechnie, Dongya Y. Yang

**Affiliations:** 1 Ancient DNA Laboratory, Department of Archaeology, Simon Fraser University, Burnaby, British Columbia, Canada; 2 Department of Anthropology, University of Victoria, Victoria, British Columbia, Canada; 3 Hakai Institute, Heriot Bay, Quadra Island, British Columbia, Canada; National Cheng Kung University, TAIWAN

## Abstract

Rockfish (*Sebastes* spp.) are a common marine fish in nearshore and continental shelf environments in the North Pacific Ocean. They are frequently identified in coastal archaeological sites in western North America; however, the morphological similarity of rockfish species limits conventional zooarchaeological identifications to the genus level. This study applies ancient DNA analysis to 96 archaeological rockfish specimens from four sites on separate islands in an archipelago on western Vancouver Island, British Columbia, Canada. Two of the archaeological sites are located within a marine protected area specifically designed to facilitate the recovery of inshore rockfish populations; two sites are located outside this boundary and remain subject to considerable fishing pressure. Using mitochondrial 16S and control region DNA sequences, we identify at least twelve different rockfish species utilized during the past 2,500 years. Identification of rockfish at closely spaced and contemporaneously occupied sites confirms that a variety of *Sebastes* species were consistently exploited at each site, with more exposed areas having a higher number of species present. Identification results indicate that four of the twelve species did not occur within the conservation area boundary and, instead, were found in sites where commercial and recreational fishing continues to be permitted. This study demonstrates that ancient DNA identifications of archaeological assemblages can complement and expand perspective on modern day fisheries conservation and management in this National Park Reserve and First Nations ancestral territory.

## Introduction

Rockfish (*Sebastes* spp.) are a diverse genus of marine fishes, with over 100 species worldwide and at least 70 in the Northeast Pacific [1]. Occupying a wide range of coastal habitats, rockfish occur at depths from the nearshore to over 1,000 metres on the continental shelf edge and sea mounts. These long-lived, predominantly non-migratory fish form mixed species assemblages that are key fixtures in nearshore marine food webs [1, 2]. The high number of species in the genus indicates a high degree of genetic diversity [3], and even relatively recent events such as late Pleistocene glaciations and corresponding sea level changes have measurably impacted the evolutionary dispersal history of certain populations [4].

Large scale industrial fisheries have targeted rockfish throughout the 20^th^ century; in the year 2000, up to 10% of annual catch in Canadian waters was rockfish [5]. Retrospective analyses of industrial and recreational fisheries documented that at the peak of industrial fishing efforts, rockfish were undergoing rapid and widespread population declines [6], which were exacerbated by their non-migratory life history and late age-at-maturity [7]. As a result, at least seven species have been classified as overfished by the U.S. National Marine Fisheries Service [8]. In areas of industrial trawl harvest, rockfish are often managed as a single, homogenous unit which can further deplete vulnerable, less abundant species [9]. Reductions in the average size of rockfish and catch per unit effort continue to be observed in areas of British Columbia [10] and may relate to high mortality from barotrauma [11] that occurs during recreational fishing for salmon, halibut, and lingcod.

Conservation area management measures have been instituted throughout the Northeast Pacific region, including the establishment of ‘no-take’ marine protected areas in Canada and the United States since the early 2000s [1, 9, 12, 13]. These no-take reserves reflect an effort to restore and sustain a variety of rockfish populations wherein large numbers of juvenile rockfish can recruit to areas of productive habitat, disperse, and recruit to nearby habitat [14, 15]. Because many rockfish species have small home ranges [16, 17] and spatially overlapping habitats, implementation of no-take conservation areas have the potential to protect multiple rockfish species simultaneously [13]. Current trends indicate that the implementation of a network of small conservation areas have not significantly influenced the recovery of rockfish [18] and violations of fishing within conservation area boundaries persist [19, 20].

As in contemporary fisheries, rockfish also played a vital component in ancient Indigenous fisheries throughout the Pacific Coast. A recent meta-analysis compiling 40 years of research on archaeological fisheries in the region indicates that rockfish are present in over 60% of archaeological sites on the Northwest Coast, and are the fifth most commonly occurring fish taxa in sites from Oregon to southeast Alaska [21]. Rockfish are particularly abundant at archaeological sites along the exposed outer coast where high relief rocky reef habitats predominate [21, 22].

The widespread archaeological occurrence of rockfish can aid fisheries management and conservation by connecting modern and ancient observations, thereby extending ecological baselines and contextualizing how modern populations may have shifted since the onset of industrial harvesting [23]. However, conventional species identification of archaeological rockfish remains is not possible due to morphological similarities of skeletal elements between species. As a result, archaeological studies of rockfish have remained limited to genus level analyses [21, 24]. Here we use ancient DNA to identify the archaeological composition of harvested rockfish species to better characterize past human fishing practices in an area of the Pacific Northwest Coast. This study aims to contribute to the increasingly broad range of research that combines Indigenous knowledge and archaeological data with marine protected area management to enhance fisheries conservation and marine planning efforts [24–29].

### Study area

**Fig 1:**
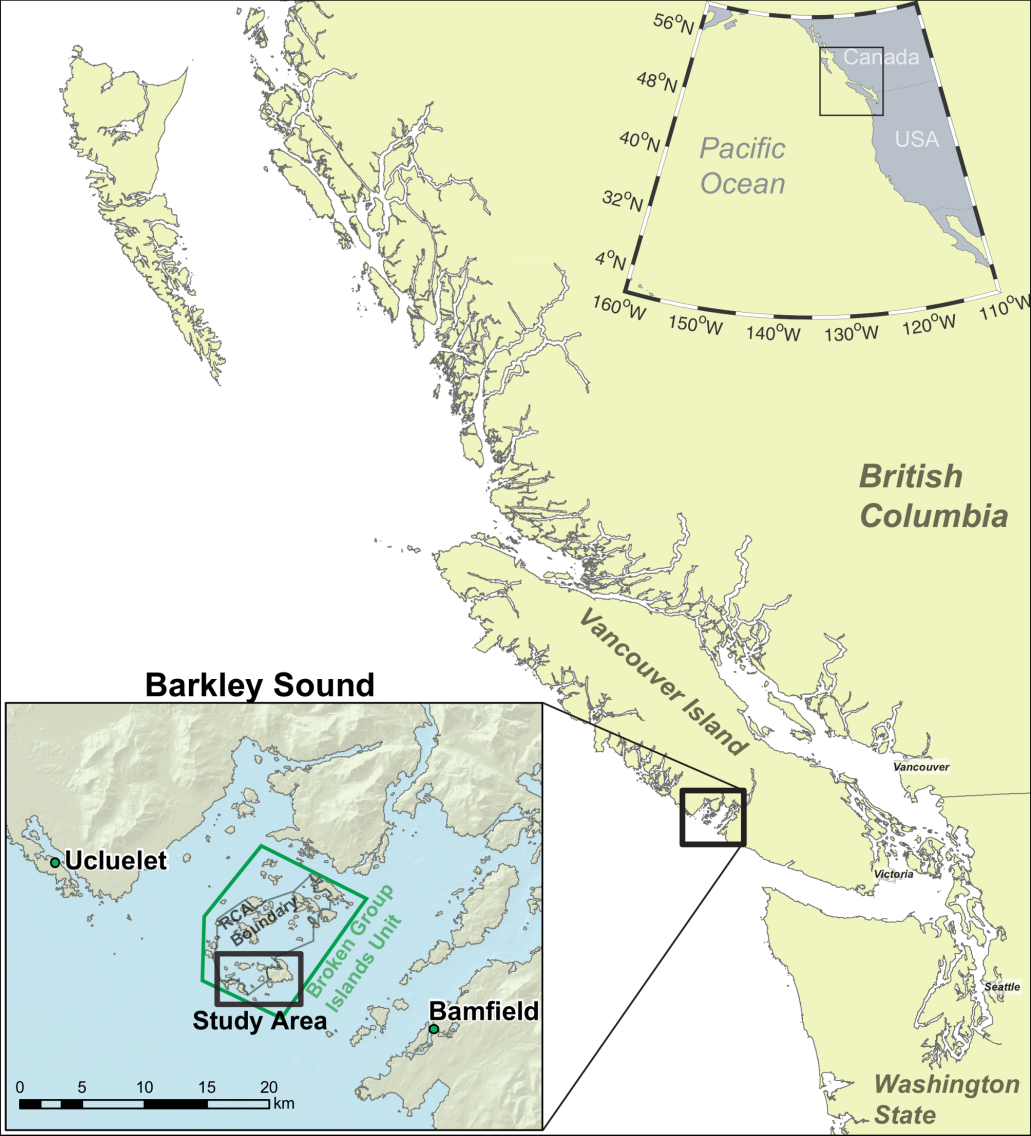
Overview of study area from which rockfish bones were recovered and analyzed.

The geographic focus of this study is the Broken Group Island archipelago in Barkley Sound on the west coast of Vancouver Island, British Columbia, Canada (Fig 1). Indigenous peoples have occupied the British Columbia coast for at least 13,000 years [30, 31], intensively utilizing the marine environment as evidenced by the thousands of shell midden habitation sites (anthropogenic sediments containing evidence of past harvesting effort) documented throughout the region [32]. At the time of contact with Europeans (AD 1774), Indigenous communities on western Vancouver Island (Nuu-chah-nulth peoples) occupied densely-populated and strongly defined territories, including intertidal and offshore fishing grounds [33–35]. The presence of over 40 large shell midden settlements (> 3000 m^2^) and historic accounts [36, 37] indicate that the population of Barkley Sound is estimated to have been 8,500 people (approximately double the contemporary human population in this region) [38–40]. At least five politically autonomous Nuu-chah-nulth groups controlled Barkley Sound during the mid 19^th^ century, but many more independent groups were present prior to a series of contact-era tribal amalgamations that occurred during waves of introduced diseases [36, 38, 39, 41]. Nuu-chah-nulth communities depended economically on the marine environment, and fishing and shellfishing were among the most frequently and widely practiced subsistence activities [33, 35, 41].

The Broken Group Island archipelago is located within the recognized traditional territory of the Tseshaht First Nation (a member of the Nuu-chah-nulth Tribal Council). Since 1971, it has been part of the federally managed Broken Group Islands ‘Unit’ of the Pacific Rim National Park Reserve (Parks Canada). In 2004, approximately 42% of this unit of the National Park Reserve was designated a rockfish conservation area (RCA) by Canada’s Department of Fisheries and Oceans [42]. This targeted marine conservation status aimed to restrict the removal of inshore rockfish by commercial and recreational fishers inside the major islands fringing the archipelago (Fig 1). This RCA was one of many established across the British Columbia coast designed to facilitate the recovery of inshore rockfish populations [12, 20, 42].

## Materials and methods

### Archaeological samples

The archaeological rockfish remains analyzed in this study were recovered from late-Holocene archaeological contexts in the southern portion of the Broken Group Islands, British Columbia, Canada (Fig 1). Permission to excavate at these heritage sties was provided by a Tseshaht First Nation Council resolution and Parks Canada Agency Research and Collection Permits #PRN-2008-1579 and 2009–2737 [36, 43, 44]. Zooarchaeological analysis of vertebrate remains identified 8,255 fish specimens to species, genus or family level from 366 sediment samples from seven sites representing approximately 272 litres [43:269]. Ancient DNA analysis was applied to 96 morphologically identified rockfish samples from six vertebrate assemblages from four archaeological sites (ca. 2,500–250 years BP) located within and just outside the Rockfish Conservation Area in the Broken Group Island archipelago (Fig 1; S1 Table). The rockfish skeletal samples were recovered from spatially and stratigraphically distinct levels at different settlements in order to avoid sampling multiple elements from the same individual [43]. Regression based body size estimation data from these sites indicate that relatively small (and younger) rockfish predominate (mean size = 26 cm, SD ±7 cm) [40:273] and are similar to a previously examined assemblage within 2 km of the study area [45].

### DNA extractions

DNA extraction and analysis was carried out in the dedicated Ancient DNA Laboratory at Simon Fraser University, Canada. The Ancient DNA Laboratory is specifically designed for the analysis of ancient DNA and includes UV filtered ventilation, positive airflow, bench UV lights, and dedicated equipment and reagents. No modern DNA samples have ever been processed in the lab and strict contamination control protocols are followed at all times [46]

Rockfish skeletal samples, ranging from <10–221 mg, were chemically decontaminated through submersion in 6% sodium hypochlorite (bleach) for seven minutes before being rinsed twice in ultra-pure water and UV irradiated in a cross-linker for 15–30 minutes on two sides. The samples were then manually crushed into powder and incubated overnight in 4 mL of lysis buffer [0.5 M EDTA (pH 8.0), 0.5% SDS, and 0.5 mg/mL proteinase K] in a rotating hybridization oven at 50°C. DNA was extracted using the modified silica-spin column method described by Yang et al. [47] and Speller et al. [46]

### Mitochondrial DNA analysis

Molecular species identification relies on a comprehensive set of comparative reference DNA sequences for accuracy. The recovered DNA samples were PCR amplified and sequenced for two regions of the mitochondrial genome that are useful for discriminating among *Sebastes* species and have corroborated reference DNA sequences from 101 rockfish species [3]. PCR amplifications targeted fragments totaling 575 bp for 16S rRNA and 512 bp for the control region (S2 Table). PCR amplifications were conducted in a Mastercycler Gradient (Eppendorf) in a 30 μL reaction volume containing 50 mM KCl, 10 mM Tris-HCl, 2.5 mM MgCl_2_, 0.2 mM dNTP, 1.0 mg/mL BSA, 0.3 μM each primer, 3.0–4.0 μL DNA sample and 2.25 U AmpliTaq Gold (Life Technologies Corporation, Carlsbad, CA). PCR began with an initial 12 minute denaturing period at 95°C, followed by 60 cycles at 94°C for 30 seconds (denaturing), 55°C for 30 seconds (annealing), and 72°C extension for 40 seconds. Blank extracts and negative controls were included in each PCR setup. PCR products were sequenced using both forward and reverse primers at Eurofins MWG Operon, Inc. (Huntsville, Alabama). A subset of the samples underwent replication of PCR and sequencing to check the reproducibility of the results and to detect any base pair misincorporations due to DNA damage.

The obtained ancient sequences were compared to GenBank sequences through the BLAST application (http://www.ncbi.nlm.nih.gov/BLAST/), to determine their closest match and to ensure that they did not match any other unexpected species or sequences. Sample sequences identified as *Sebastes* were visually edited and base pair ambiguities were examined using ChromasPro software (www.technelysium.com.au). Resulting edited sequences analyzed against comparative modern DNA reference sequences were approximately 521 bp and 398 bp for 16S rRNA and the control region respectively. Multiple alignments of the ancient sequences and published reference sequences were achieved using [48], through BioEdit (www.mbio.ncsu.edu). Neighbor-joining trees were constructed using Kimura’s 2-parameter model in the Mega 6.0 software program [49]. Species identifications were assigned only if a sequence matched identically or very closely with published reference sequences in a manner that was distinguishable from the next closest related species and if no other evidence, including reproducibility tests or additional markers from the same sample, indicated a different species.

## Results

### Ancient DNA-based identifications

Ancient DNA was successfully recovered for 92 of the 96 morphologically identified *Sebastes* specimens (95.8%) (S1 Table). Of those 92 successful amplifications, 81 were confirmed as belonging to the genus *Sebastes* (88%; GenBank Accessions MF179308-MF179457). Three specimens were only able to generate partial DNA fragments and, while confirmed as *Sebastes*, were unable to be identified to the species level and therefore excluded from further analyses. Two additional samples could not be distinguished between three potential *Sebastes* species and were also excluded from further analyses.

A BLASTN search identified the non-*Sebastes* samples as greenling *(Hexagrammos* spp.; n = 6), lingcod (*Ophiodon elongates*; n = 1), shiner perch (*Cymatogaster aggregata*; n = 1), Red Irish lord (*Hemilepidotus hemilepidotus*; n = 1), and surfperch (*Embiotoca* spp.; n = 1) which are also species that occur regularly in archaeological contexts in the study area [21] and the region more broadly [22, 45, 50]. One sample demonstrated non-specific amplification of human DNA after multiple failed amplifications and was excluded from analyses. Although morphological misidentifications were rare, these results demonstrate that ancient DNA can act as a valuable check to confirm morphological identifications of archaeological remains [51, 52].

The high amplification success rate of 95.8% is not surprising as archaeological sites on the Northwest Coast have proven to have exceptional DNA preservation, likely due to the fairly narrow range of temperature fluctuation, consistent precipitation, and alkalinity of shell midden sediments safeguarding skeletal remains from the wet coastal climate and acidic forest soils [47]. Similar results have been obtained from other archaeogenetic studies in the same region [46, 53].

The results of the DNA amplification and sequence analysis suggest that the recovered rockfish DNA is authentic. The contamination controls undertaken in this study were successful at eliminating any systematic contamination as no PCR amplification was observed in blank extracts and PCR negative controls. Furthermore, the use of multiple markers increased our confidence in the species identifications: in all instances that both markers successfully amplified, no identifications conflicted. However, it is important to note that while analyzing multiple genes improves species identifications, the taxonomic framework under which the species identifications are assigned also needs to be scrutinized. Species identifications were assigned by phylogenetic comparisons to modern reference sequences made available by Hyde and Vetter [3] and, when available, additional comparative sequences publicly available in GenBank. As not all of the 100+ species of rockfish receive the same attention in scientific research, majority of the rockfish species only had one comparative reference DNA sequence available. Additional genetic insights into interspecific and intraspecific variation in rockfish species can only improve the accuracy of species identification.

### Evaluating sampling effort

**Fig 2:**
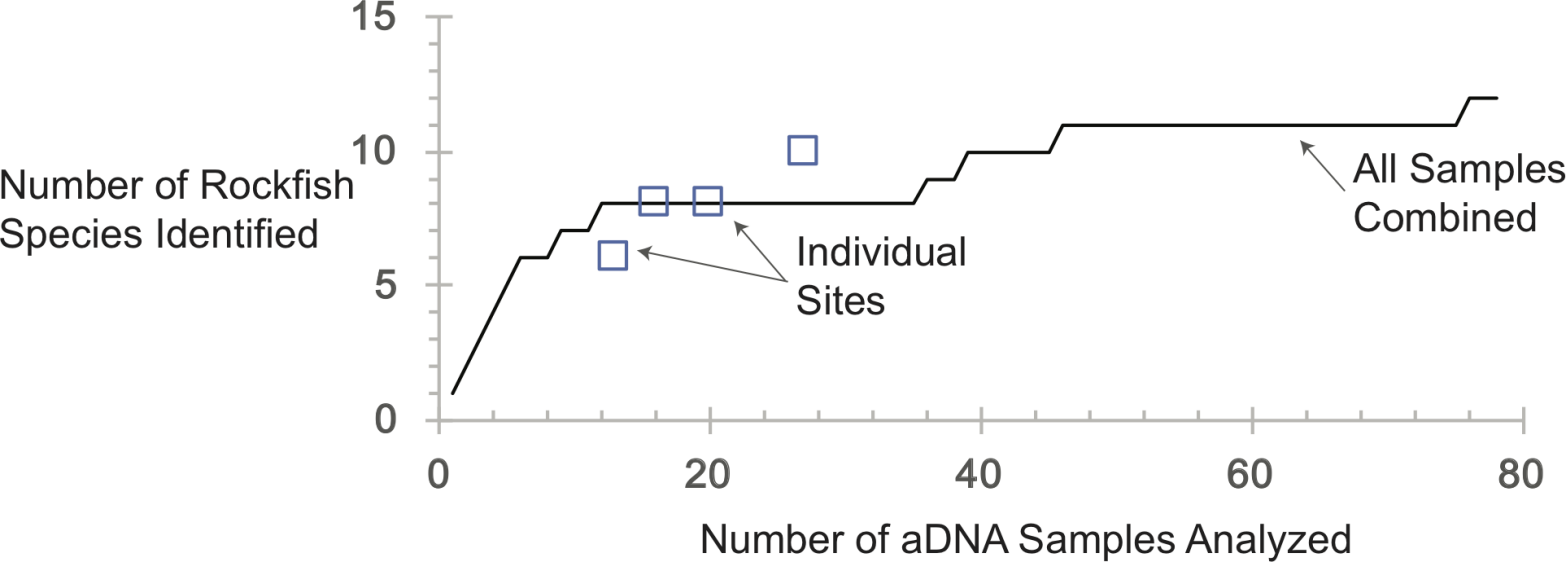
Species accumulation curve for all rockfish species analyzed.

The mathematical inevitabilities of archaeological and paleobiological sampling dictate that the more sampling, the more variety [54, 55]. To address the issue of sampling effort with ancient DNA identifications, we generated a simple species accumulation curve that demonstrates that the number of rockfish species identified increases with sample size but appears to reach an inflection point, after which the rate at which newly identified species occur diminishes (Fig 2). As expected, this analysis shows that more species were observed as more samples were examined. The bulk of species present have been detected with 15–20 samples and assemblages with the greatest number of samples analyzed also have the highest number of species detected (e.g., Effingham and Wouwer–Old, n = 16 and n = 14 respectively, with 8 species each). Given the limited resources available to conduct these analyses and the very large number of rockfish species, we do not aim to have detected the true number of species represented archaeologically, but rather to have approached redundancy in this particular sample.

### Main fishing pattern

**Fig 3:**
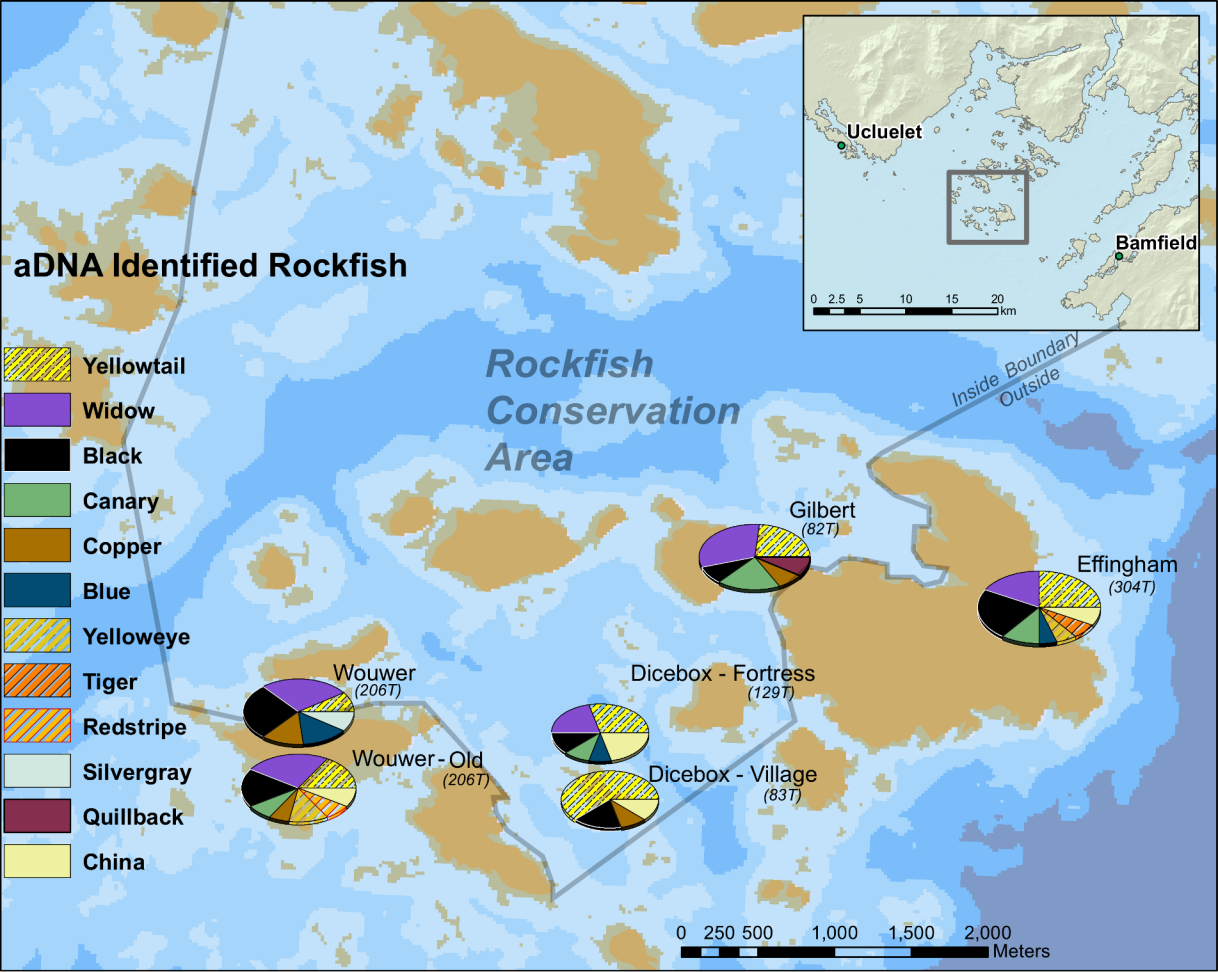
Map of the proportions of identified rockfish samples from six assemblages from four archaeological sites in the Broken Group Island archipelago.

Despite the small study area and modest sample size from each site, twelve different rockfish species were identified (Table 1; Fig 3), a number similar to contemporary ecological observations within Barkley Sound [56–58]. The broad range of rockfish species identified overall is also observed within the individual archaeological sites: eight species were identified in two of the six archaeological assemblages, and six species in an additional three assemblages (Table 1; Fig 3). The six species that occur most frequently across all sites are yellowtail (*S*. *flavidus*), widow (*S*. *entomelas*), black (*S*. *melanops*), canary (*S*. *pinniger*), china (*S*. *nebulosus*), and copper rockfish (*S*. *caurinus*).

**Table 1:** Number of identified rockfish specimens identified to species per archaeological site.

Species	Site						Total
	Dicebox Fortress	Dicebox Village	Effingham	Gilbert	Wouwer	Wouwer Old	
Yellowtail rockfish *(S*. *flavidus)*	3	6	4	3	1	2	19
Widow rockfish *(S*. *entomelas)*	2	-	3	4	4	4	17
Black rockfish *(S*. *melanops)*	1	2	3	1	3	2	12
Canary rockfish *(S*. *pinniger*)	1	-	2	3	-	1	7
China rockfish *(S*. *nebulosus*)	2	1	1	-	-	1	5
Copper rockfish *(S*. *caurinus)*	-	1	-	1	2	1	5
Blue rockfish *(S*. *mystinus)*	1	-	1	-	2	-	4
Yelloweye rockfish *(S*. *ruberrimus)*	-	-	1	-	-	2	3
Quillback rockfish *(S*. *maliger)*	-	-	-	1	-	-	1
Redstripe rockfish *(S*. *proriger)*	-	-	-	-	-	1	1
Silvergray rockfish *(S*. *brevispinis)*	-	-	-	-	1	-	1
Tiger rockfish *(S*. *nigrocinctus)*	-	-	1	-	-	-	1
**Total**	**10**	**10**	**16**	**13**	**13**	**14**	**76**

For detailed specimen identifications see S1 Table.

The observed archaeological patterning indicates an overall degree of similarity and continuity in rockfish habitat and/or fishing practice over the past 2,500 years in this exposed coastal archipelago. Potential temporal shifts in the assemblage could also reflect periods of climatic change and/or broader cultural shifts in fishing effort [59–62], but the small sample size in this study precludes such an evaluation.

**Fig 4:**
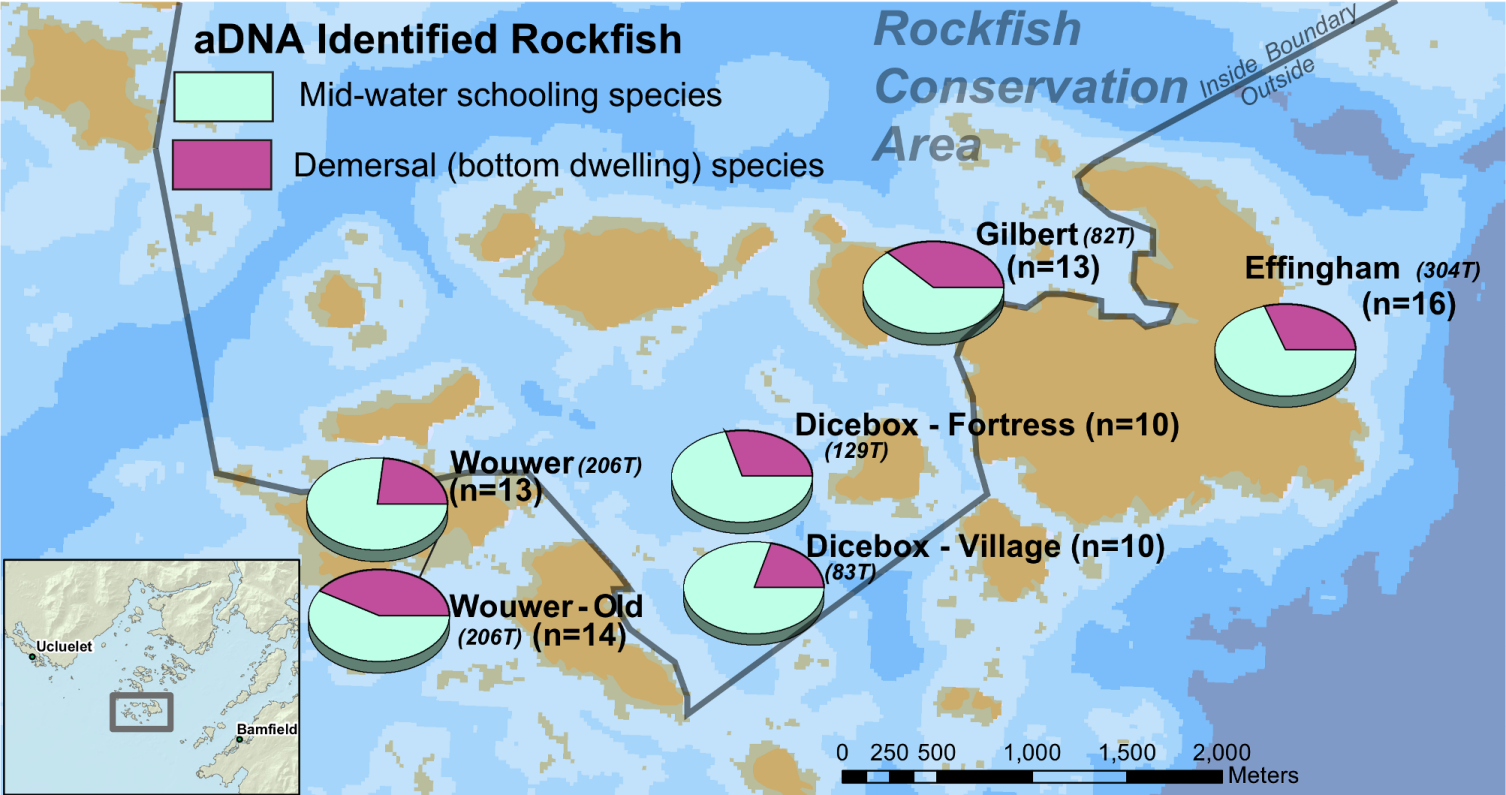
Map of the proportions of rockfish ecotypes identified at each archaeological site. Mid-water schooling species include black, blue, widow, and yellowtail rockfish. Demersal solitary species include canary, china, copper, quillback, redstripe, silvergray, tiger, and yelloweye rockfish [1].

The diverse and complex life history traits of rockfish have facilitated divergent speciation but with a high degree of spatial overlap in habitat preferences and depth ranges [18]. Some rockfish exhibit preference for schooling in mid-water kelp forests; some have crevice-associated bottom dwelling preferences; and some are associated with the continental slope edge in very deep waters [1, 2]. The majority of archaeological rockfish identified in this study are composed of mid-water schooling rockfish (62–80%); the remaining species are solitary rockfish associated with demersal habitats (Fig 4). The rank order of these two rockfish ‘ecotypes’ exhibit consistent patterning across all sites and time periods. This patterning indicates that the bulk of rockfish fishing effort targeted mid-water schooling fish associated with kelp habitat. For example, yellowtail (n = 19) and black (n = 12) rockfish are present in all six assemblages, and widow rockfish (n = 17) is present in five (Table 1). Furthermore, yellowtail, widow, and black rockfish make up a large proportion of species present in all six archaeological assemblages (57%-80%), indicating a ubiquitous utilization of these three species.

There remains variation within and between archaeological sites, with the more recent deposits at Dicebox and Wouwer containing the highest percentages (77–80%) of mid-water schooling rockfish (Table 1). In contrast, the site with the greatest number of crevice dwelling demersal fish is also the oldest in the group (Wouwer ca. 2,500–1,200 years BP). The two archaeological assemblages with the greatest number of rockfish species were also in the most swell exposed coastal settings (Effingham and Wouwer–Old), where both exposure and kelp habitat predominate. Such broad similarities in species composition across sites and slight environmental and temporal differences between sites indicates that fishing effort targeted specific locations in close proximity to settlements.

Many of the shallow reefs, channels, and islets in close proximity to the study sites contain highly productive kelp habitat. The distribution of kelp can vary significantly seasonally with exposure and current speeds, as well as with the presence or absence of sea otters in the region [63]. Sea otters have dramatic yet indirect ecological effects on the abundance of rockfish as they help regulate sea urchins which graze kelp forests to a small percentage of their potential size and depth range (reducing the habitat available for rockfish). Accordingly, rockfish recruitment and biomass can be much higher when sea otters are present and considerably lower in areas where sea otters are absent [63]. As a result, the long-term ecological consequences of sea otters being removed from the west coast of Vancouver Island during the maritime fur trade (ca. AD 1778–1803) may have resulted in a potential large-scale reduction in rockfish habitat relative to what was prevalent during the pre-contact time period. Further exploration of this hypothesis is warranted with stable isotopes and other proxy data [64–66], but even if lower numbers were present, the study area remains highly suitable habitat where abundance is expected to vary due to localized climactic effects, as well as the amount of kelp present [59, 67].

## Discussion

This study is the first investigation of ancient DNA from archaeological rockfish and affirms the long history of human use of rockfish in this modern-day marine protected area. By extending the ecological baseline past the industrial fishing era, these results pertain to contemporary marine conservation efforts focused on rockfish species in this same archipelago and elsewhere on the west coast of North America. Connecting these efforts and observations across large time scales helps contextualize and evaluate historical change, as well as the suitability of ongoing conservation and restoration efforts [27].

This study has identified twelve rockfish species that were harvested within close proximity to each archaeological site location, both inside and outside a contemporary marine protected area, in a region that remains the focus of intensive commercial and recreational fishing effort [19]. The wide range of species identified reveals that numerous rockfish species were culturally desired and a broadly targeted food resource. Their persistent abundance in the zooarchaeological record relative to other fish indicates that millennia of Indigenous fishing practices have not appeared to have negatively affected rockfish abundance in the Broken Group Islands [43, 45, 68].

As inshore rockfish are generally non-migratory, they could be obtained year-round and were likely critical to predictable provisioning for the dense Indigenous communities living in large settlements throughout the archipelago and surrounding areas of Barkley Sound [43, 69, 70]. Despite a vulnerability to overharvesting, rockfish clearly withstood millennia of intensive harvest practices. The sustainability of these harvests was likely considerably enhanced and enabled by the territorially specific marine tenure systems that were present historically in this archipelago [38], as well as more broadly in Nuu-chah-nulth communities on western Vancouver Island [33] and across the Northwest Coast [71]. Locally, such tenure systems featured highly specific fishing territories, including named islands, islets, and reefs that designated family and local group boundaries [39]. These boundaries were broadly known and rigidly enforced by individuals and lineage-based household groups, and are highly compatible with modern conservation efforts of spatial management [72] that guide and inform current rockfish conservation efforts [10, 73].

### Conservation implications

Understanding the history of rockfish fisheries and identifying potential mismatches in rockfish conservation management strategies is especially relevant considering the vulnerability of rockfish to overharvesting given their longevity (maximum ages range from 50–120 years for the species studied here) and long generation times [74]. The contemporary focus on harvesting large, reproductively powerful, older rockfish is particularly detrimental as it removes large fecund females that have a disproportionate contribution to recruitment, limiting recovery and future population growth [6, 7, 75]. The modern trend of catching larger, older rockfish contrasts with archaeological rockfish size indicating smaller individuals were targeted in this area in the past [45] potentially enabling a higher overall harvest rate.

**Fig 5:**
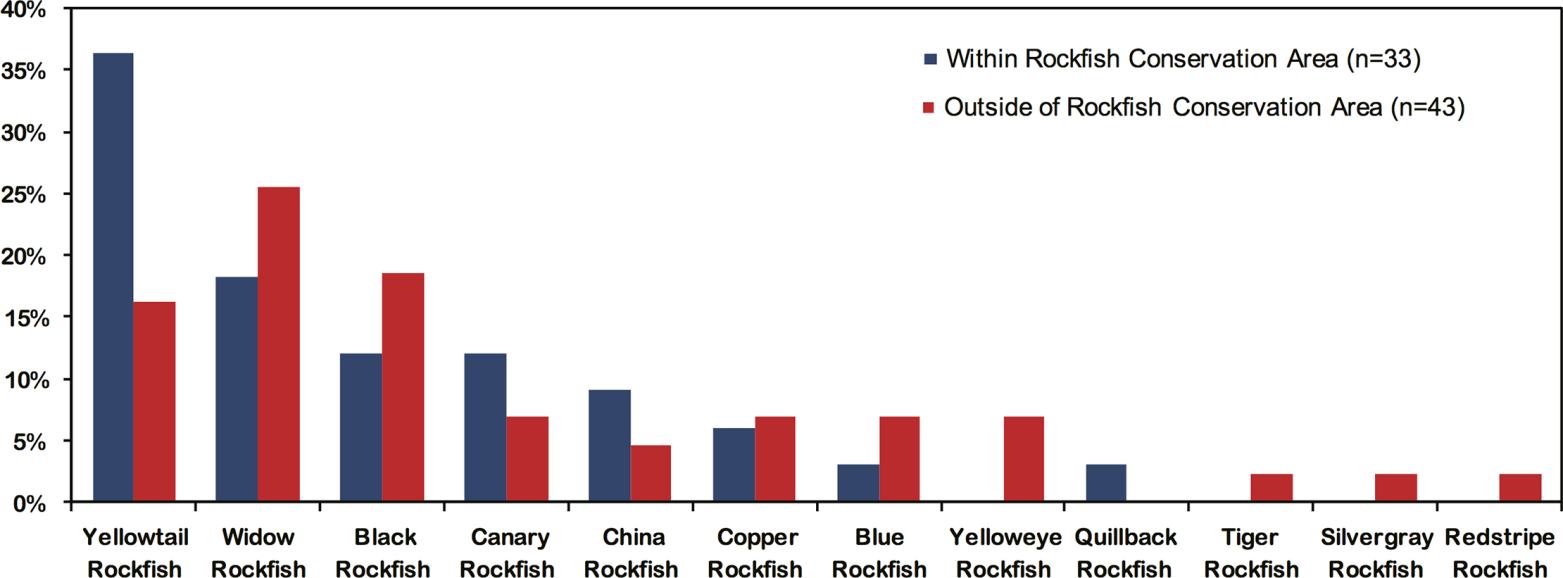
Proportion of archaeological rockfish species detected within and immediately outside of the rockfish conservation area in the Broken Group Island archipelago.

The presence and proportions of rockfish species found within and immediately outside of a marine protected area enable the comparison of species being included and/or overlooked by such spatial management measures (Fig 5). Since 2004, the interior portions of the Broken Group Island archipelago have been managed as a modern day conservation area where hook and line commercial fishing is not permitted [42]. Three of the six archaeological assemblages examined in this study are situated immediately outside the conservation area; the other three assemblages are located within the boundary. Based on a range of other zooarchaeological evidence discussed previously, it is likely that the majority of rockfish were obtained in close proximity to each site.

A key observation is that four of the twelve detected rockfish species (redstripe, silvergray, yelloweye, and tiger rockfish) were not identified in sites located inside the rockfish conservation area (Fig 5). While this may be influenced in part by low overall sampling effort, it introduces the possibility that up to 30% of the species traditionally harvested by Indigenous peoples in this archipelago may not be protected by this conservation area boundary. This includes yelloweye rockfish, which can live over 120 years and are currently listed as a species of “special concern” in Canada [76]. Similarly, tiger rockfish with a maximum recorded age of 116 years, is only observed at sites outside the conservation area. Both species were among the five inshore species for which these protected areas were originally designed to specifically target for conservation [12]. It is notable that these and other species continue to be subject to considerable commercial and recreational and recreational fishing effort [19, 77]. In contrast, only one of the five original target species (quillback rockfish) is exclusively present within the rockfish conservation area. The remaining species (china and copper rockfish) are found at multiple sites both within and outside the protected area boundary.

### Conclusion

This case study demonstrates that ancient DNA analysis of archaeological fisheries data can broaden insight for understanding rockfish conservation on the Pacific Coast of North America. Archaeogenetic data can help shed light on the long-term biogeography of species, with the potential to recognize reductions in species abundance and track genetic diversity over time. While there were over twelve individual yellowtail, widow, and black rockfish detected among the ancient samples (n = 19, n = 17, and n = 12 respectively), the small sample sizes prevent meaningful population genetic analyses, as does a lack of comparable modern DNA sequence data. Future studies will benefit from examining additional archaeological sites and samples of ancient and modern rockfish. The examination of larger and more spatially and temporally distributed samples sizes will provide valuable information on the biogeographic distribution and genetic structure of rockfish species, and extend the baseline data on species distributions and abundances.

DNA was found to be well preserved in as little as <10 mg of archaeological skeletal material, a result consistent with previous studies on archaeological herring samples from the same region [46]. Based on the results of this study and ongoing work on herring, considerable potential exists for recovering nuclear DNA from the archaeological rockfish remains, giving a clearer picture of the historic population structure within rockfish and other fish species. For rockfish, this would provide a unique line of evidence that may aid stock-specific management, as some rockfish species show fine-scale population structure, and others show evidence of widespread gene flow [8]. By combining the time depth of archaeology with the precision of genetic analyses, a clearer ecological picture will emerge for management decisions, potentially allowing for the harvesting of abundant stocks and/or species, while protecting depleted stocks and/or species.

## Supporting information

S1 Table Species identification of the analyzed archaeological rockfish samples. “PF” denotes a partial DNA fragment of the target region was amplified. “No amp” denotes no DNA amplification. “Undetermined” indicates that the marker could not distinguish between multiple rockfish species.
(DOCX)

S2 Table 16S and control region *Sebastes* mtDNA PCR amplification primers. (DOCX)

## References

[1] Love MS, Yoklavich M, Thorsteinson LK. The rockfishes of the Northeast Pacific. Berkeley: University of California Press; 2002 x, 404 p.

[2] Gunderson DR, Vetter RD. Temperate rocky reef fishes In: Sale PF, Kritzer JP, editors. Marine Metapopulations. San Diego: Academic Press; 2006 p. 69–117.

[3] Hyde JR, Vetter RD. The origin, evolution, and diversification of rockfishes of the genus *Sebastes* (Cuvier). Molecular Phylogenetics and Evolution. 2007;44:790–811. doi: 10.1016/j.ympev.2006.12.026 17320419 10.1016/j.ympev.2006.12.026

[4] Buonaccorsi VP, Kimbrell CA, Lynn EA, Vetter RD. Population structure of copper rockfish (*Sebastes caurinus*) reflects postglacial colonization and contemporary patterns of larval dispersal. Canadian Journal of Fisheries and Aquatic Sciences. 2002;59:1374–84.

[5] Beamish RJ, Benson AJ, Sweeting RM, Neville CM. Regimes and the history of the major fisheries of Canada’s west coast. Progress in Oceanography. 2004;60:355–85.

[6] Levin PS, Holmes EE, Piner KR, Harvey CJ. Shifts in a Pacific Ocean fish assemblage: The potential influence of exploitation. Conservation Biology. 2006;20(4):1181–90. 16922234 10.1111/j.1523-1739.2006.00400.x

[7] Berkeley SA, Hixon MA, Larson RJ, Love MS. Fisheries sustainability via protection of age structure and spatial distribution of fish populations. Fisheries. 2004;29:23–32.

[8] Berntson EA, Levin PS, Moran P. Conservation of North Pacific rockfishes: Ecological genetics and stock structure. Proceedings of the Workshop March 2–3, 2004 Seattle, Washington. Seattle: Northwest Fisheries Science Center, Conservation Biology Division, National Marine Fisheries Service, 2007.

[9] Gunderson DR. Spatial Patterns in the dynamics of slope rockfish stocks and their implications for management. Fishery Bulletin. 1997;95:219–30.

[10] McGreer M, Frid A. Declining size and age of rockfishes (*Sebastes* spp.) inherent to Indigenous cultures of Pacific Canada. Ocean & Coastal Management. 2017;145:14–20.

[11] Pribyl AL, Schreck CB, Kent ML, Parker SJ. The differential response to Decompression in three species of nearshore Pacific rockfish. North American Journal of Fisheries Management. 2009;29(5):1479–86.

[12] Yamanaka KL, Logan G. Developing British Columbia's inshore rockfish conservation strategy. Marine and Coastal Fisheries. 2010;2(1):28–46.

[13] Paddack MJ, Estes JA. Kelp forest fish populations in marine reserves and adjacent exploited areas of central California. Ecological Applications. 2000;10(3):855–70.

[14] Lotterhos KE, Markel RW. Oceanographic drivers of offspring abundance may increase or decrease reproductive variance in a temperate marine fish Molecular Ecology. 2012;21(20):5009–26. doi: 10.1111/j.1365-294X.2012.12002.x 22978484 10.1111/j.1365-294X.2012.12002.x

[15] Lotterhos KE, Dick SJ, Haggarty DR. Evaluation of rockfish conservation area networks in the United States and Canada relative to the dispersal distance for black rockfish (*Sebastes melanops*). Evolutionary Applications. 2014;7(2):238–59. doi: 10.1111/eva.12115 24567745 10.1111/eva.12115 PMC3927886

[16] Marliave J, Frid A, Welch DW, Porter AD. Home site fidelity in black rockfish, *Sebastes melanops*, reintroduced into a fjord environment. Canadian Field Naturalist. 2013;127:255–61.

[17] Mathewes K. A telemetric study of the home ranges and homing routes of copper and quillback rockfishes on shallow rocky reefs. Canadian Journal of Zoology. 1990;68:2243–50.

[18] Haggarty DR, Shurin JB, Yamanaka KL. Assessing population recovery inside British Columbia’s Rockfish Conservation Areas with a remotely operated vehicle. Fisheries Research. 2016;183:165–79.

[19] Haggarty DR, Martell S, Shurin JB. Lack of recreational fishing compliance may compromise effectiveness of Rockfish Conservation Areas in British Columbia. Canadian Journal of Fisheries and Aquatic Sciences. 2016;73:1587–98.

[20] Lancaster D, Dearden P, Ban NC. Drivers of recreational fisher compliance in temperate marine conservation areas: A study of Rockfish Conservation Areas in British Columbia, Canada. Global Ecology and Conservation. 2015;4(7):645–57.

[21] McKechnie I, Moss ML. Meta-analysis in zooarchaeology expands perspectives on Indigenous fisheries of the Northwest Coast of North America. Journal of Archaeological Science: Reports. 2016;8:470–85.

[22] Orchard TJ. Late Holocene fisheries in Gwaii Haanas: Species composition, trends in abundance, and environmental or cultural explanations In: Moss ML, Cannon A, editors. The Archaeology of North Pacific Fisheries. Fairbanks: University of Alaska Press; 2011 p. 111–28.

[23] Al-Abdulrazzak D, Naidoo R, Palomares MLD, Pauly D. Gaining perspective on what we've lost: The reliability of encoded anecdotes in historical ecology. PLoS One. 2012;7(8):e43386 doi: 10.1371/journal.pone.0043386 22937043 10.1371/journal.pone.0043386 PMC3427348

[24] Braje TJ, Rick T, Erlandson JM. Rockfish in the long view: Applied zooarchaeology and conservation of Pacific red snapper (Genus *Sebastes*) in Southern California In: Wolverton S, Lyman RL, editors. Conservation Biology and Applied Zooarchaeology. Tucson: University of Arizona Press; 2012 p. 157–78.

[25] Eckert LE, Ban NC, Frid A, McGreer M. Diving back in time: Extending historical baselines for yelloweye rockfish with Indigenous knowledge Aquatic Conservation: Marine and Freshwater Ecosystems 2017; doi: 10.1002/aqc.2834

[26] Armstrong CG, Shoemaker A, McKechnie I, Ekblom A, Szabó P, Lane PJ, et al Anthropological contributions to historical ecology: 50 Questions, infinite prospects. PLoS One. 2017;12(2):e0171883 doi: 10.1371/journal.pone.0171883 28235093 10.1371/journal.pone.0171883 PMC5325225

[27] Ban NC, Kittinger JN, Pandolfi JM, Pressey RL, Thurstan RH, Lybolt MJ, et al Incorporating historical perspectives into systematic marine conservation planning In: Kittinger JN, McClenachan L, Gedan KB, Blight LK, editors. Marine Historical Ecology in Conservation: Applying the Past to Manage for the Future. Berkeley: University of California Press; 2015 p. 207–33.

[28] Quintana Morales EM, Lepofsky D, Berkes F. Ethnobiology and fisheries: Learning from the past for the present. Journal of Ethnobiology. 2017;37(3):369–79.

[29] Ban NC, Frid A. Indigenous Peoples' rights and Marine Protected Areas. Marine Policy. 2018;87:180–5.

[30] McLaren D, Rahemtulla F, Gitla (Elroy White), Fedje DW. Prerogatives, Sea level and the strength of persistent places: Archaeological evidence for long-term occupation of the Central Coast of British Columbia. BC Studies. 2015;187:155–91.

[31] Mackie Q, Fedje DW, McLaren D, Smith NF, McKechnie I. Early environments and archaeology of Coastal British Columbia In: Bicho NF, Haws JA, Davis LG, editors. Trekking the Shore: Changing Coastlines and the Antiquity of Coastal Settlement. New York: Springer; 2011 p. 51–103.

[32] McMillan AD, McKechnie I. Investigating Indigenous adaptations to British Columbia’s exposed outer coast: Introduction to these outer shores. BC Studies. 2015;(187):3–20.

[33] McMillan AD. Since the time of the transformers: The Ancient heritage of the Nuu-chah-nulth, Ditidaht, and Makah. Vancouver: UBC Press; 1999.

[34] Mackie Q. Settlement archaeology in a fjordland archipelago: Network analysis, social practice, and the built environment of Western Vancouver Island, British Columbia, Canada since 2000 BP. Oxford: British Archaeological Reports; 2001.

[35] Drucker P. The Northern and Central Nootkan Tribes. Washington D.C.: Smithsonian Institution; 1951.

[36] McKechnie I. Indigenous oral history and settlement archaeology in Barkley Sound, Western Vancouver Island. BC Studies. 2015;(187):191–225.

[37] Haggarty JC, Inglis RI. Historical resources site survey and assessment, Pacific Rim National Park. Ottawa: Report submitted to National Historic Parks and Sites Branch, Parks Service, 1985.

[38] Claire DE St.. Barkley Sound Tribal Territories In: Arima EY, Claire DE St., Clamhouse L, Edgar J, Jones C, Thomas J, editors. Between Ports Alberni and Renfrew: Notes on West Coast Peoples. Mercury Series. 121. Ottawa: Canadian Ethnology Service, Canadian Museum of Civilization; 1991 p. 13–202.

[39] McMillan AD, Claire DE St.. Ts'ishaa: Archaeology and ethnohistory of a Nuu-chah-nulth origin site in Barkley Sound. Carlson R, editor. Burnaby: Archaeology Press, Simon Fraser University; 2005.

[40] Clayton DW. Islands of Truth: The imperial fashioning of Vancouver Island. Vancouver: UBC Press; 2000 xxii, 330 p.

[41] McMillan AD, McKechnie I, Claire DE St., Frederick SG. Exploring variability in maritime resource use on the Northwest Coast: A case study from Barkley Sound, Western Vancouver Island. Canadian Journal of Archaeology. 2008;32(2):214–38.

[42] Department of Fisheries and Oceans Canada. Rockfish Conservation Areas: Protecting British Columbia's rockfish [Online booklet]. Ottawa: Fisheries and Oceans Canada, Pacific Region; 2006 [updated January 10, 2007]. Available from: http://www.pac.dfo-mpo.gc.ca/recfish/Restricted_Areas/RCAs/booklet/default_e.htm.

[43] McKechnie I. An archaeology of food and settlement on the Northwest Coast [PhD Dissertation]. Vancouver: University of British Columbia; 2014.

[44] McKechnie I. Archaeological research in the Broken Group Islands, Pacific Rim National Park Reserve, June 2009. Interim Report Submitted to: Tseshaht First Nation, Port Alberni, BC; Pacific Rim National Park Reserve of Canada, Ucluelet, BC; and Parks Canada Cultural Resource Services, Western and Northern Service Centre, Victoria, BC, 2010.

[45] McKechnie I. Investigating the complexities of sustainable fishing at a prehistoric village on Western Vancouver Island, British Columbia, Canada. Journal for Nature Conservation. 2007;15(3):208–22.

[46] Speller CF, Hauser L, Lepofsky D, Peterson D, Moore J, Rodrigues A, et al High Potential for using DNA from ancient herring Bones to inform modern fisheries management and conservation. PLoS One. 2012;7(11):e51122 doi: 10.1371/journal.pone.0051122 23226474 10.1371/journal.pone.0051122 PMC3511397

[47] Yang DY, Cannon A, Saunders SR. DNA Species identification of archaeological salmon bone from the Pacific Northwest Coast of North America. Journal of Archaeological Science. 2004;31(5):619–31.

[48] Thompson JD, Higgins DG, Gibson TJ. CLUSTAL W: Improving the sensitivity of progressive multiple sequence alignment through sequence weighting, position-specific gap penalties and weight matrix choice. Nucleic Acids Research. 1994;22(22):4673–80. 7984417 10.1093/nar/22.22.4673 PMC308517

[49] Tamura K, Stecher G, Peterson D, Filipski A, Kumar S. MEGA6: Molecular evolutionary genetics analysis version 6.0. Molecular Biology and Evolution. 2013;30(12):2725–9. doi: 10.1093/molbev/mst197 24132122 10.1093/molbev/mst197 PMC3840312

[50] Szpak P, Orchard TJ, Salomon AK, Gröcke DR. Regional ecological variability and impact of the maritime fur trade on nearshore ecosystems in Southern Haida Gwaii (British Columbia, Canada): Evidence from stable isotope analysis of rockfish (*Sebastes* spp.) bone collagen. Archaeological and Anthropological Sciences. 2013;5(2):159–82.

[51] Nims R, Butler VL. Assessing reproducibility in faunal analysis using blind tests: A case study from northwestern North America. Journal of Archaeological Science: Reports. 2017;11(2):750–61.

[52] Wolverton S. Data quality in zooarchaeological faunal identification. Journal of Archaeological Method and Theory. 2013;20(3):381–96.

[53] Moss ML, Yang DY, Newsome SD, Speller CF, McKechnie I, McMillan AD, et al Historical ecology and biogeography of North Pacific pinnipeds: Isotopes and ancient DNA from three archaeological assemblages. Journal of Island and Coastal Archaeology. 2006;1(2):165–90.

[54] Lepofsky D, Lertzman KP. More on sampling for richness and diversity in archaeobiological assemblages. Journal of Ethnobiology. 2005;25(2):175–88.

[55] Lyman RL, Ames KM. Sampling to redundancy in zooarchaeology: Lessons from the Portland Basin, Northwestern Oregon and Southwestern Washington. Journal of Ethnobiology. 2004;24(2):329–46.

[56] Markel RW. Rockfish recruitment and trophic dynamics on the West Coast of Vancouver Island: Fishing, ocean climate, and sea otters [PhD Dissertation]. Vancouver: University of British Columbia; 2011.

[57] Leaman BM. The ecology of fishes in British Columbia kelp beds I. Barkley Sound *Nereocystis* beds. Fisheries Development Report No. 22, Marine Research Branch, Province of British Columbia, 1980.

[58] Holmes H, Tomascik T, editors. Underwater visual surveys of inshore groundfish with particular focus on rockfish, Broken Group Islands, Pacific Rim National Park Reserve. Fifth Annual International Conference of Science and the Management of Protected Areas; 2003; University of Victoria, Victoria, British Columbia.

[59] Markel RW, Lotterhos KE, Robinson CKL. Temporal variability in the environmental and geographic predictors of spatial-recruitment in nearshore rockfishes. Marine Ecology Progress Series. 2017;574:97–111.

[60] Monks GG. Evidence of changing climate and subsistence strategies among the Nuu-chah-nulth of Canada’s West Coast In: Monks GG, editor. Climate Change and Human Responses: A Zooarchaeological Perspective. Dordrecht: Springer; 2017 p. 173–96.

[61] McKechnie I. Zooarchaeological analysis of the Indigenous fishery at the Huu7ii Big House and Back Terrace, Huu-ay-aht Territory, Southwestern Vancouver Island In: McMillan AD, Claire DE St., editors. Huu7ii: Household Archaeology at a Nuu-chah-nulth Village Site in Barkley Sound. Burnaby, BC: Archaeology Press, Simon Fraser University; 2012 p. 154–86.

[62] McKechnie I. Appendix E: Column sampling and the archaeology of small fish at Ts'ishaa In: McMillan AD St. Claire DE, editors. *Ts'ishaa*: Archaeology and Ethnography of a Nuu-chah-nulth Origin Site in Barkley Sound. Burnaby: Archaeology Press, Simon Fraser University; 2005 p. 206–23.

[63] Markel RW, Shurin JB. Indirect effects of sea otters on rockfish (*Sebastes* spp.) in giant kelp forests. Ecology. 2015;96(11):2877–90. 27070008 10.1890/14-0492.1

[64] Ramshaw BC, Pakhomov EA, Markel RW, Kaehler S. Quantifying spatial and temporal variations in phytoplankton and kelp isotopic signatures to estimate the distribution of kelp-derived detritus off the west coast of Vancouver Island, Canada. Limnology and Oceanography. 2017; doi: 10.1002/lno.10555

[65] Szpak P, Orchard TJ, McKechnie I, Gröcke DR. Historical ecology of Late Holocene sea otters (*Enhydra lutris*) from Northern British Columbia: Isotopic and zooarchaeological perspectives. Journal of Archaeological Science. 2012;39(5):1553–71.

[66] Singh GG, Markel RW, Martone RLG, Salomon AK, Harley CDG, Chan KMA. Sea otters homogenize mussel beds and reduce habitat provisioning in a rocky intertidal ecosystem. PLoS One. 2013;8(5):e65435 doi: 10.1371/journal.pone.0065435 23717697 10.1371/journal.pone.0065435 PMC3663835

[67] Haggarty DR, Lotterhos KE, Shurin JB. Young-of-the-year recruitment does not predict the abundance of older age classes in black rockfish in Barkley Sound, British Columbia, Canada. Marine Ecology Progress Series. 2017;574:113–26.

[68] Frederick G, Crockford SJ. Appendix D: Analysis of the vertebrate fauna from Ts'ishaa village, DfSi 16, Benson Island In: McMillan AD St. Claire DE, editors. Ts'ishaa: Archaeology and Ethnography of a Nuu-chah-nulth Origin Site in Barkley Sound. Burnaby: Archaeology Press, Simon Fraser University; 2005 p. 173–205.

[69] Monks GG. Locational optimization and faunal remains in Northern Barkley Sound, Western Vancouver Island, British Columbia In: Moss ML, Cannon A, editors. The Archaeology of North Pacific Fisheries. Fairbanks: University of Alaska Press; 2011 p. 129–48.

[70] McKechnie I. Five thousand years of fishing at a shell midden in the Broken Group Islands, Barkley Sound, British Columbia [MA Thesis]. Burnaby: Simon Fraser University; 2005.

[71] Trosper RL. Northwest Coast Indigenous institutions that supported resilience and sustainability. Ecological Economics. 2002;41(2):329–44.

[72] Ban NC, Picard C, Vincent ACJ. Moving toward spatial solutions in marine conservation with Indigenous communities. Ecology and Society. 2008;13(1):32.

[73] Frid A, McGreer M, Haggarty DR, Beaumont J, Gregr EJ. Rockfish size and age: The crossroads of spatial protection, central place fisheries and Indigenous rights. Global Ecology and Conservation. 2016;8:170–82.

[74] Williams GD, Levin PS, Palsson WA. Rockfish in Puget Sound: An ecological history of exploitation. Marine Policy. 2010;34(5):1010–20.

[75] Berkeley SA, Chapman C, Sogard SM. Maternal age as a determinant of larval growth and survival in a marine fish, *Sebastes melanops*. Ecology. 2004;85(5):1258–64.

[76] Department of Fisheries & Oceans Canada. Stock assessment for the outside population of yelloweye rockfish (*Sebastes ruberrimus*) for British Columbia, Canada in 2014. Canadian Scientific Advisory Secretariat Stock Status Report 2015/060, 2015.

[77] Ban NC, Alidina HM, Ardron JA. Cumulative impact mapping: Advances, relevance and limitations to marine management and conservation, using Canada's Pacific waters as a case study. Marine Policy. 2010;34(5):876–86.

