# Supplementary material for: Ancient DNA analysis of Indigenous rockfish use on the Pacific Coast: Implications for marine conservation areas and fisheries management
Source: PLoS One. 2018 Feb 13;13(2):e0192716. doi: 10.1371/journal.pone.0192716 (PMC5811035; doi:10.1371/journal.pone.0192716)
Supplement: S1 Table — “PF” denotes a partial DNA fragment of the target region was amplified. “No amp” denotes no DNA amplification. “Undetermined” indicates that the marker could not distinguish between multiple rockfish species. (DOCX) [file pone.0192716.s001.docx]

**S1 Table. Species identification of the analyzed archaeological rockfish samples.** “PF” indicates a partial fragment of the target region was amplified. “No amp” denotes no DNA amplification. “Undetermined” indicates that the marker could not distinguish between multiple rockfish species.

| **Specimen** | **Archaeological**  **Site** | **Provenience** | **16S ID** | **Control Region ID** | **Species ID** |
| --- | --- | --- | --- | --- | --- |
| RF05 | Dicebox Fortress | 129T6J8 | China | China | China  *(S. nebulosus*) |
| RF22 | Dicebox Fortress | 129T2E2 | Canary | Canary | Canary  *(S. pinniger*) |
| RF24 | Dicebox Fortress | 129T2E7 | Black, Olive or Yellowtail | Yellowtail | Yellowtail  *(S. flavidus)* |
| RF26 | Dicebox Fortress | 129T3D2 | *Sebastes* spp. (PF) | No amp | Undetermined  (*Sebastes* spp.) |
| RF29 | Dicebox Fortress | 129T2D5 | Greenling | No amp | Mis ID  (Not *Sebastes* spp.) |
| RF30 | Dicebox Fortress | 129T3C2 | Black, Olive or Yellowtail | Yellowtail | Yellowtail  *(S. flavidus)* |
| RF39 | Dicebox Fortress | 129T9C1 | China | No amp | China  *(S. nebulosus*) |
| RF53 | Dicebox Fortress | 129T2E3 | Blue or Widow | Widow | Widow  *(S. entomelas)* |
| RF70 | Dicebox Fortress | 129T6A2C | *Sebastes* spp. (PF) | Blue | Blue  *(S. mystinus)* |
| RF71 | Dicebox Fortress | 129T6B3C | Black, Olive or Yellowtail | Yellowtail | Yellowtail  *(S. flavidus)* |
| RF72 | Dicebox Fortress | 129T6J9 | Black, Olive or Yellowtail | No amp | Black, Olive or Yellowtail  (*S. melanops/serranoides/flavidus)* |
| RF73 | Dicebox Fortress | 129T3E3 | Black, Olive or Yellowtail | No amp | Black, Olive or Yellowtail  (*S. melanops/serranoides/flavidus)* |
| RF74 | Dicebox Fortress | 129T2D5 | Greenling | No amp | Mis ID  (Not *Sebastes* spp.) |
| RF75 | Dicebox Fortress | 129T2E4 | Black, Olive or Yellowtail | Black | Black  *(S. melanops)* |
| RF76 | Dicebox Fortress | 129T2F2 | No amp | No amp | No amp |
| RF77 | Dicebox Fortress | 129T2C2 | No amp | No amp | No amp |
| RF78 | Dicebox Fortress | 12T18B3 | Blue or Widow | Widow | Widow  *(S. entomelas)* |
| RF02 | Dicebox Village | 83TWBF | Black, Olive or Yellowtail | Yellowtail | Yellowtail  *(S. flavidus)* |
| RF14 | Dicebox Village | 83T1D5 | China | China | China  *(S. nebulosus*) |
| RF15 | Dicebox Village | 83T10A5 | Black, Olive or Yellowtail | Yellowtail | Yellowtail  *(S. flavidus)* |
| RF16 | Dicebox Village | 83T1D3 | Greenling | No amp | Mis ID  (Not *Sebastes* spp.) |
| RF18 | Dicebox Village | 83T5C5 | Black, Olive or Yellowtail | Black | Black  *(S. melanops)* |
| RF19 | Dicebox Village | 83T5C11 | Lingcod | Lingcod | Mis ID  (Not *Sebastes* spp.) |
| RF38 | Dicebox Village | 83T11A3 | *Sebastes* spp. (PF) | No amp | Undetermined  (*Sebastes* spp.) |
| RF44 | Dicebox Village | 83T7B7 | Black, Olive or Yellowtail | Yellowtail | Yellowtail  *(S. flavidus)* |
| RF51 | Dicebox Village | 83T10A2 | Black, Olive or Yellowtail | Yellowtail | Yellowtail  *(S. flavidus)* |
| RF79 | Dicebox Village | 83T5C6 | Black, Olive or Yellowtail | Black | Black  *(S. melanops)* |
| RF80 | Dicebox Village | 83T5C4 | Black, Olive or Yellowtail | Yellowtail | Yellowtail  *(S. flavidus)* |
| RF81 | Dicebox Village | 83T7A5 | Irish lord | No amp | Mis ID  (Not *Sebastes* spp.) |
| RF82 | Dicebox Village | 83T7C2 | *Sebastes* spp. (PF) | No amp | Undetermined  (*Sebastes* spp.) |
| RF83 | Dicebox Village | 83T11A6 | Black, Olive or Yellowtail | Yellowtail | Yellowtail  *(S. flavidus)* |
| RF84 | Dicebox Village | 83T12A7 | Non-specific amplification | No amp | Non-specific  amplification |
| RF85 | Dicebox Village | 83T12A5 | Undetermined | Copper | Copper  *(S. caurinus)* |
| RF01 | Effingham | 304T3C11a | Blue or Widow | Blue | Blue  *(S. mystinus)* |
| RF09 | Effingham | 304T4A18 | Canary | Canary | Canary  *(S. pinniger*) |
| RF21 | Effingham | 304T1A2 | Black, Olive or Yellowtail | Black | Black  *(S. melanops)* |
| RF27 | Effingham | 304T4A19 | Blue or Widow | Widow | Widow  *(S. entomelas)* |
| RF28 | Effingham | 30T4A13 | Black, Olive or Yellowtail | Black | Black  *(S. melanops)* |
| RF31 | Effingham | 304T20B3 | Black, Olive or Yellowtail | Yellowtail | Yellowtail  *(S. flavidus)* |
| RF32 | Effingham | 304T1A9 | Black, Olive or Yellowtail | Yellowtail | Yellowtail  *(S. flavidus)* |
| RF37 | Effingham | 304T4A12 | *Sebastes* spp. (PF) | Black | Black  *(S. melanops)* |
| RF45 | Effingham | 304T4A17 | Undetermined | Tiger | Tiger  *(S. nigrocinctus)* |
| RF47 | Effingham | 304T4A14 | Blue or Widow | Widow | Widow  *(S. entomelas)* |
| RF55 | Effingham | 304T1A10 | Blue or Widow | Widow | Widow  *(S. entomelas)* |
| RF63 | Effingham | 304T4A6 | Yelloweye | Yelloweye | Yelloweye  *(S.* ruberrimus) |
| RF64 | Effingham | 304T4A11 | Greenling | No amp | Mis ID  (Not *Sebastes* spp.) |
| RF65 | Effingham | 304T4A23 | Black, Olive or Yellowtail | Yellowtail | Yellowtail  *(S. flavidus)* |
| RF66 | Effingham | 304T1A3 | Black, Olive or Yellowtail | Yellowtail | Yellowtail  *(S. flavidus)* |
| RF67 | Effingham | 304T1A7 | Shiner perch | No amp | Mis ID  (Not *Sebastes* spp.) |
| RF68 | Effingham | 304T1A8 | China | China | China  *(S. nebulosus*) |
| RF69 | Effingham | 304T3C6 | Canary | Canary | Canary  *(S. pinniger*) |
| RF04 | Gilbert | 82T9A2 | Black, Olive or Yellowtail | Black | Black  *(S. melanops)* |
| RF10 | Gilbert | 82T5A8 | Undetermined | Quillback | Quillback  *(S. maliger)* |
| RF23 | Gilbert | 82T9A4 | Blue or Widow | Widow | Widow  *(S. entomelas)* |
| RF25 | Gilbert | 82T9A12 | Greenling | No amp | Mis ID  (Not *Sebastes* spp.) |
| RF36 | Gilbert | 82T5A6 | Blue or Widow | Widow | Widow  *(S. entomelas)* |
| RF40 | Gilbert | 82T5A12 | Canary | Canary | Canary  *(S. pinniger*) |
| RF43 | Gilbert | 82T9A6 | Canary | Canary | Canary  *(S. pinniger*) |
| RF48 | Gilbert | 82T9A9 | Canary | Canary | Canary  *(S. pinniger*) |
| RF49 | Gilbert | 82T4A5 | Black, Olive or Yellowtail | Yellowtail | Yellowtail  *(S. flavidus)* |
| RF57 | Gilbert | 82T4A8 | Blue or Widow | Widow | Widow  *(S. entomelas)* |
| RF58 | Gilbert | 82T9A10 | Black, Olive or Yellowtail | Yellowtail | Yellowtail  *(S. flavidus)* |
| RF59 | Gilbert | 82T9A5 | Undetermined | Copper | Copper  *(S. caurinus)* |
| RF60 | Gilbert | 82T9A3 | Black, Olive or Yellowtail | Yellowtail | Yellowtail  *(S. flavidus)* |
| RF61 | Gilbert | 82T4A1 | Greenling | No amp | Mis ID  (Not *Sebastes* spp.) |
| RF62 | Gilbert | 82T4A8 | Blue or Widow | Widow | Widow  *(S. entomelas)* |
| RF07 | Wouwer | 206T4B12 | Blue or Widow | Widow | Widow  *(S. entomelas)* |
| RF08 | Wouwer | 206T5B9 | Black, Olive or Yellowtail | Black | Black  *(S. melanops)* |
| RF11 | Wouwer | 206T4B5 | Blue or Widow | Widow | Widow  *(S. entomelas)* |
| RF12 | Wouwer | 206T4E15 | Undetermined | Copper | Copper  *(S. caurinus)* |
| RF17 | Wouwer | 206T4B11 | Blue or Widow | Widow | Widow  *(S. entomelas)* |
| RF33 | Wouwer | 206T4B35 | Blue or Widow | Blue | Blue  *(S. mystinus)* |
| RF35 | Wouwer | 206T4B13 | Black, Olive or Yellowtail | Black | Black  *(S. melanops)* |
| RF41 | Wouwer | 206T20B5 | Black, Olive or Yellowtail | Black | Black  *(S. melanops)* |
| RF46 | Wouwer | 206T5B5 | Black, Olive or Yellowtail | Yellowtail | Yellowtail  *(S. flavidus)* |
| RF52 | Wouwer | 206T20B11 | Undetermined | Silvergray | Silvergray  *(S. brevispinis)* |
| RF86 | Wouwer | 206T4B11 | Blue or Widow | Blue | Blue  *(S. mystinus)* |
| RF87 | Wouwer | 206T4B9 | Blue or Widow | Widow | Widow  *(S. entomelas)* |
| RF88 | Wouwer | 206T4B24 | Undetermined | Copper | Copper  *(S. caurinus)* |
| RF06 | Wouwer Old | 206T9E20 | Blue or Widow | Widow | Widow  *(S. entomelas)* |
| RF13 | Wouwer Old | 206T9E2 | China | China | China  *(S. nebulosus*) |
| RF20 | Wouwer Old | 206T9E4 | Blue or Widow | Widow | Widow  *(S. entomelas)* |
| RF34 | Wouwer Old | 206T9E14 | Black, Olive or Yellowtail | Black | Black  *(S. melanops)* |
| RF42 | Wouwer Old | 206T9C1 | *Sebastes* spp. (PF) | Yelloweye | Yelloweye  *(S.* ruberrimus) |
| RF50 | Wouwer Old | 206T9D10 | Blue or Widow | Widow | Widow  *(S. entomelas)* |
| RF54 | Wouwer Old | 206T12D4 | Canary | Canary | Canary  *(S. pinniger*) |
| RF56 | Wouwer Old | 206T9E17 | Black, Olive or Yellowtail | Yellowtail | Yellowtail  *(S. flavidus)* |
| RF89 | Wouwer Old | 206T9E3 | Undetermined | Copper | Copper  *(S. caurinus)* |
| RF90 | Wouwer Old | 206T9E12 | Black, Olive or Yellowtail | Black | Black  *(S. melanops)* |
| RF91 | Wouwer Old | 206T9E16 | Black, Olive or Yellowtail | Yellowtail | Yellowtail  *(S. flavidus)* |
| RF92 | Wouwer Old | 206T9D11 | Surfperch | No amp | Mis ID  (Not *Sebastes* spp.) |
| RF93 | Wouwer Old | 206T9E18 | Yelloweye | Yelloweye | Yelloweye  *(S.* ruberrimus) |
| RF94 | Wouwer Old | 206T9C3 | No amp | No amp | No amp |
| RF95 | Wouwer Old | 206T9D2 | Undetermined | Redstripe | Redstripe  *(S. proriger)* |
| RF96 | Wouwer Old | 206T9D4 | No amp | No amp | No amp |
| RF97 | Wouwer Old | 206T9D10 | Blue or Widow | Widow | Widow  *(S. entomelas)* |
