# Supplementary material for: Ancient DNA analysis of Indigenous rockfish use on the Pacific Coast: Implications for marine conservation areas and fisheries management
Source: PLoS One. 2018 Feb 13;13(2):e0192716. doi: 10.1371/journal.pone.0192716 (PMC5811035; doi:10.1371/journal.pone.0192716)
Supplement: S2 Table — (DOCX) [file pone.0192716.s002.docx]

**S2 Table. 16S and control region *Sebastes* mtDNA PCR amplification primers.**

| **Amplified Region** | **Primer** | **Sequence (5'-3')** | **Reference** |
| --- | --- | --- | --- |
| 16S | 16SA | CGCCTGTTTATCAAAAACAT | Palumbi et al. [78] |
| 16S | 16SA-2 | CYGYRGCTCTTMRYTGTAGGAGKG | This study |
| 16S | 16SB-2 | KCCCTAAYGTCTTTGGTTGGGGC | This study |
| 16S | 16SB | CCGGTCTGAACTCAGATCACGT | Palumbi et al. [78] |
| Control Region | D-RF | CCTGAAAATAGGAACCAAATGCCAG | Hyde and Vetter [3] |
| Control Region | Thr-RF | GAGGAYAAAGCACTTGAATGAGC | Hyde and Vetter [3] |
